# Antiviral Activity of Allyl Isothiocyanate Against Infectious Viruses

**DOI:** 10.1007/s12560-025-09643-5

**Published:** 2025-05-20

**Authors:** Irene Falcó, Gloria Sánchez

**Affiliations:** 1 Department of Microbiology and Ecology, University of Valencia, Av. Dr. Moliner, 50., 46100 Burjassot, Valencia Spain; 2 Environmental Virology and Food Safety Lab (VISAFELab), Department of Preservation and Food Safety Technologies, Institute of Agrochemistry and Food Technology, IATA-CSIC, Av. Agustín Escardino 7, 46980 Paterna, Valencia Spain

**Keywords:** Enteric viruses, Allyl isothiocyanate, Natural compounds

## Abstract

The effects of allyl isothiocyanate (AITC) on enteric viruses, specifically hepatitis A virus (HAV) and murine norovirus (MNV) as a norovirus surrogate, were evaluated at different concentrations, temperatures, and exposure time. AITC at 0.1 and 0.5% was mixed with each virus and incubated at 10, 25, and 37 °C for 2 h or overnight. AITC demonstrated a concentration-, temperature-, and time-dependent antiviral effect, with the lowest concentration resulting in a modest decrease in viral titer. However, at the highest concentration and 37 °C during overnight incubation, reductions of 3.75 log TCID50/mL for MNV and below the limit of detection for HAV were reported. Additionally, efficacy of AITC was evaluated on human norovirus (HuNoV) GI suspensions using an in situ capture quantitative reverse transcription polymerase chain reaction (RT-qPCR) method. The results indicated that HuNoVs are susceptible to AITC at 37 °C, which partially inhibits the interaction between the viral capsid and its receptor. Furthermore, AITC was tested as a natural disinfectant for produce with treatment times of 15 and 30 min, with no statistically significant changes in viral titers. Although further optimization of AITC application is required, these findings suggest that AITC has potential as a tool to reduce enteric virus contamination on food and food-contact surfaces.

## Introduction

Foodborne viruses are widely recognized as the leading cause of reported outbreaks in industrialized countries (EFSA & CDC, 2023; Harrison & DiCaprio, 2018). Among these, human enteric viruses are the most frequently identified etiological agents, particularly in produce-related outbreaks, accounting for 54% of cases, which are often associated with improper food handling practices (Bennett et al., 2018).

Notably, norovirus was the third most frequently reported causative agent of foodborne outbreaks in Europe in 2022, with an 11.6% increase in human cases compared to 2021. Specifically, there were 7,305 cases of norovirus, representing 15.0% of all outbreak-associated cases that year. This virus was responsible for 12 major epidemics across multiple Member States, each involving more than 100 cases, and exhibited a high mean outbreak size of 22.0 cases per outbreak (EFSA & CDC, 2023).

While food may spread a wide range of viruses, the most commonly reported include HuNoV, astrovirus (HAstV), and rotavirus A (RV), all of which cause gastroenteritis (Carter, 2005), and hepatitis A and E viruses (HAV/HEV), whose symptoms include weakness, sudden nausea and vomiting, stomachache (especially near the liver), loss of appetite, fever, and headache (Arguedas & Fallon, 2004). Contamination sources include food items like leafy greens, berries, and shellfish, as well as food handlers, equipment, and surfaces (fomites) that may harbor viral particles, facilitating secondary transmission in food-related environments (Abad et al., 2001; Sánchez et al., 2015). The primary transmission route for enteric viruses is fecal–oral. These viruses can contaminate surfaces, remain infectious on food-contact surfaces and food products for weeks (Croci et al., 2002; Mattison et al., 2007), and infect the host with a very low infectious dose (10–100 viral particles) (Teunis et al., 2008). This persistence, combined with the low infectious dose, significantly increases the risk of viral outbreaks from contaminated food products.

Since 2013, the World Health Organization (WHO) has been encouraging the development of alternative processing technologies to ensure food microbiological safety (WHO, 2013). In this context, generally recognized as safe (GRAS) compounds have gained increasing interest from the food industry, consumers, and researchers, particularly for their suitability as natural antimicrobials in food applications. Moreover, these compounds are aligned with the growing trend of green consumerism, which favors the use of natural products, offering a more affordable alternative to chemically synthesized antibacterials and antivirals (Burt, 2004). While the antimicrobial properties of many natural products against bacteria and fungi are well documented, there is limited research on their antiviral applications in food.

Hydrolytic products, such as isothiocyanates (ITCs), have been reported to be effective against bacteria, fungi, and insects (Agrawal & Kurashige, 2003; Müller et al., 2018; Singh et al., 2022). Notably, allyl isothiocyanate (AITC) holds four active Environmental Protection Agency (EPA) registrations and is used in the US as an insecticide, bactericide, and nematicide (Singh et al., 2022). AITC is a key active compound found in cruciferous vegetables like broccoli, kale, and horseradish (Kegley, 2000; Kuang et al., 2013). Numerous investigations have demonstrated AITC’s anticancer properties and its strong broad-spectrum antibacterial activity, including effectiveness against *Listeria monocytogenes* and *Salmonella typhimurium* (Chacon et al., 2006; Jiao et al., 1996; Kuang et al., 2013; Liang et al., 2006). In addition to inhibiting bacterial growth, AITC also exhibits antifungal properties. For example, it has been shown to prevent the germination and growth of *Penicillium expansum*, *Aspergillus flavus*, and *Botrytis cinerea* conidia (Kleszczyński et al., 2013).

In the present work, the effect of AITC on the infectivity of HAV and murine norovirus (MNV), a cultivable human norovirus surrogate, was investigated. Additionally, the antiviral activity of AITC against human norovirus GI (HuNoV) was assessed using an in situ capture RT-qPCR (ISC-RT-qPCR) method based on porcine gastric mucin binding. Furthermore, its potential application as a natural disinfectant for vegetables was assessed.

## Materials and Methods

### Clinical Samples, Cell Lines, and Virus Propagation

A fecal sample containing human (HuNoV genogroup I genotype 4 (kindly provided by Dr. Buesa, University of Valencia, Spain) was suspended (10%, wt/vol) in PBS containing 2 M NaNO_3_ (Panreac), 1% beef extract (Conda), and 0.1% Triton X-100 (Fisher Scientific) at pH 7.2. The mixture was vortexed and then centrifuged at 1000×*g* for 5 min. The supernatant was aliquoted and stored at − 80 °C.

MNV-1 was propagated and assayed in RAW 264.7 cells (both kindly provided by Prof. H. W. Virgin, Washington University School of Medicine, USA), while HAV strain HM-175/18f (purchased from ATCC VR-1402) was propagated in FRhK-4 cells (purchased from ATCC VR-1688)). Virus stocks were produced by infecting their respective cell lines for 2 days for MNV and 12 days for HAV, followed by three freeze–thaw cycles and centrifugation at 660×*g* for 30 min. Infectious viruses were quantified by determining the 50% tissue culture infectious dose (TCID_50_) using the Spearman–Karber method (Falcó et al., 2023).

### Antiviral Activity of Allyl Isothiocyanate in Suspension

MNV and HAV suspensions (approximately 6 and 5 log TCID_50_/mL, respectively) were mixed in equal volumes with AITC (purity > 95%, Merck-Sigma) at concentrations of 0.1 (pH 7.1) or 0.5% (pH 7.4). The solutions were incubated at 10, 25 or 37 °C during 16 h (overnight, ON) or 2 h in a bath-shakerat100 rpm. Reactions were neutralized by adding Dulbecco´s Modified Eagle Medium (DMEM) with 10% fetal calf serum (FCS) as recommended in ISO 14476 (ISO 14476, 2015). Viruses were recovered, and tenfold dilutions of the samples were inoculated into confluent cell lines in 96-well plates. Infectious viruses were the enumerated by cell culture assays as described above. The decay of MNV and HAV titers was calculated as log_10_ (N_t_/N_0_), where N_0_ is the infectious virus titer for the untreated samples and Nt is the infectious virus titer for the AITC-treated samples (Pintó et al., 1994). Positive controls were virus suspensions incubated without AITC but with PBS under the same experimental conditions. Each treatment was performed in triplicate.

### Binding of Human Norovirus GI to Porcine Gastric Mucin

To elucidate the antiviral activity of AITC against HuNoV, a fecal suspension of HuNoV GI was treated with an equal volume of 0.5% AITC ON at 37 °C. Samples were processed by *in-situ* capture RT-qPCR (ISC-RT-qPCR) based on porcine gastric mucin (PGM), as previously reported by Wang et al. (2014), with modifications described by Falcó et al. (2018). In brief, each well was coated with 100 μl of type III porcine gastric mucin (PGM, Sigma Aldrich) at 100 μg/ml in carbonate-bi-carbonate buffer (pH 9.6) at 37 °C for 1 h and then incubated ON at 4 °C. Simultaneously, suspensions of HuNoV GI were mixed with 0.5% AITC ON at 37 °C.

After being washed 5 times with 300 μl of PBS containing 0.05% Tween 20 and 0.3% BSA (PBSTB), the wells were blocked with 300 μl of 3% BSA in PBS at 37 °C for 2 h. The wells were washed 5 times with PBSTB, and 100 μl of HuNoV-AITC samples and controls were added to the microplate and incubated at 37 °C for 1 h. Finally, after washing 5 times with PBSTB, each well was added with 100 μl of lysis buffer from NucleoSpin® RNA virus kit (Macherey–Nagel GmbH & Co.).

Then, viral RNA was extracted using the same kit according to the manufacturer's instructions. RNA samples were analyzed in duplicate by RT-qPCR using the RNA UltraSense One-Step quantitative RT-PCR system (Invitrogen) and the set of primers and probe recommended by the ISO 15216 (ISO 15216-1, 2017) using the LightCycler 480 instrument (Roche Diagnostics, Germany). A standard curve for HuNoV GI was generated by amplifying tenfold dilutions of viral RNA by RT-qPCR in quintuplicates, and the numbers of PCRU were calculated. Control samples included HuNoV GI suspensions that either received no AITC treatment or were heated at 99 °C for 5 min. The quantification from the control HuNoV suspension (non-AITC treatment) was considered 100% binding, and the percentage of binding for the treated HuNoVs was calculated accordingly. Each sample was analyzed in triplicate, and the mean values and standard deviations (SD) were calculated.

### Allyl Isothiocyanate as a Natural Disinfectant for Lettuce And Spinach

Following the procedure described by Su and D’Souza (2013), locally purchased fresh lettuce (*Lactuca sativa* L.) and spinach (*Spinacia oleracea*) were cut into 2 × 2 cm pieces. To prevent contamination, leaves were first treated with UV light for 10 min. Thereafter, 50 µl of MNV or HAV suspensions, containing approximately 5 log TCID_50_/mL, were inoculated onto the surface of the leaves. The samples were dried under a laminar flow hood at room temperature (RT) until droplet suspensions were completely dry. Then, 100 µl of either PBS (control) or AITC solutions at concentrations of 0.1 or 0.5% were applied to the inoculated samples for 15 or 30 min at RT. To stop the reaction, 900 µl of DMEM supplemented with 10% FCS was added to the vegetable surfaces, allowing for the recovery of the viruses. The infectious viruses and the effectiveness of the treatments were calculated as described above.

### Data Statistics

Statistical analysis was carried out by the post hoc Tukey’s method (*p* < 0.05) to compare and determine differences between controls and treatments across the different assays (XLSTAT, Addinsoft SARL).

## Results

### Antiviral Efficacy of Allyl Isothiocyanate Against MNV and HAV

**Fig. 1:**
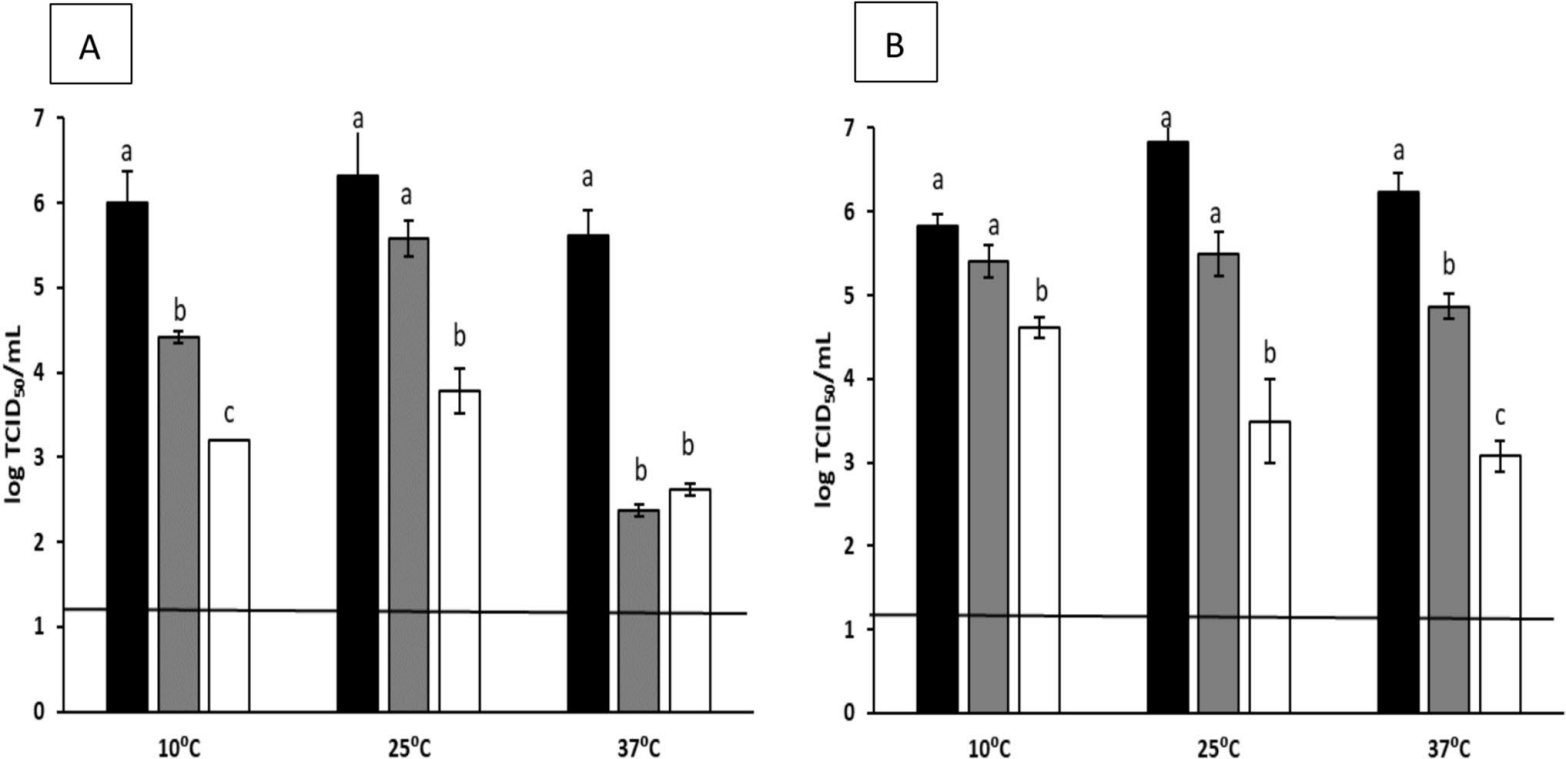
Reduction of murine norovirus (MNV) titers (log TCID_50_/mL) after overnight treatment (**A**) and 2 h treatment (**B**) with AITC at concentrations of 0.1% or 0.5% at different temperatures (10, 25, or 37 °C). Each bar represents the average of triplicates. Within each column, different letters indicate significant differences between treatments. Black bars: control; gray bars: AITC 0.1%; white bars: AITC 0.5%; black line depicts the detection limit

**Fig. 2:**
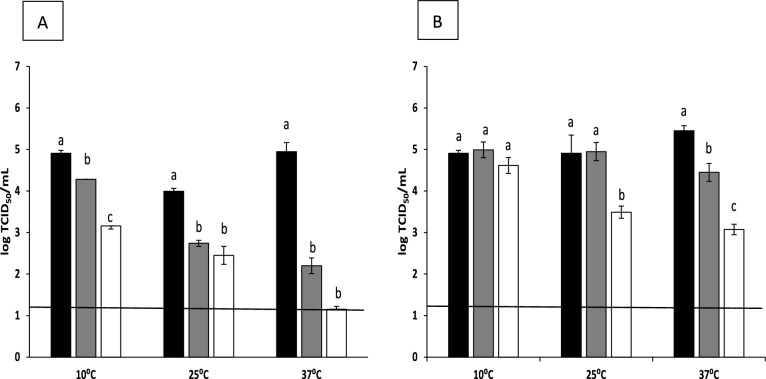
Reduction of hepatitis A virus (HAV) titers (log TCID_50_/mL) after overnight treatment (**A**) and 2 h treatment (**B**) with AITC at concentrations of 0.1% or 0.5% at different temperatures (10, 25, or 37 °C). Each bar represents the average of triplicates. Within each column, different letters indicate significant differences between treatments. Black bars: control; gray bars: AITC 0.1%; white bars: AITC 0.5%; black line depicts the detection limit

This study demonstrated the potential antiviral activity of AITC, showing a time, temperature, and concentration-dependent trend. Figures 1 and 2 show the decreasing titers of MNV and HAV, respectively, with significant differences (*p* < 0.05) observed in all samples treated with AITC at 0.5%, except for HAV treated at 10 °C after 2 h of incubation.

At a concentration of 0.1%, AITC reduced MNV titers by 1.58, 0.75, and 3.25 log when treated at 10, 25, and 37 °C, respectively. Meanwhile, 0.5% AITC reduced MNV titers by 2.79, 2.54, and 3.00 log after ON incubation (Fig. 1A). For the 2 h treatments, MNV titers decreased by 0.42, 1.33, and 1.42 log at 0.1% and 1.21, 3.33, and 3.17 log at 0.5%, 10, 25, and 37ºC, respectively (Fig. 1B).

Overall, HAV inactivation showed a similar profile (Fig. 2). Treatments with AITC at 0.1% after ON incubation resulted in reductions of 0.62, 1.25, and 2.75 log at 10, 25, and 37ºC, respectively. In contrast, AITC at 0.5% led to decreases of 1.75, 1.54, and 3.80 log at the same temperatures. When the incubation time was shortened to 2 h (Fig. 2B), HAV infectivity was reduced by 0.08, 0.04, and 1.00 log for the lowest concentration of AITC, and by 0.29, 1.42, and 2.37 log for the highest concentration at 10, 25, and 37 °C, respectively. No cytotoxic effect from AITC was observed throughout the microscopic observation.

### ISC-RT-qPCR

**Fig. 3:**
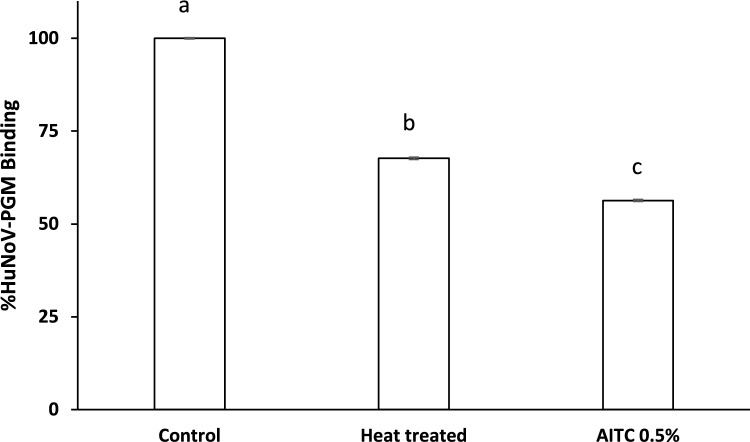
Effect of AITC (0.5%) at 37 °C after ON incubation on the binding of human norovirus GI to porcine gastric mucin (PGM) as measured by ISC-RT-qPCR. Each bar represents the average of triplicate measurements. Different letters within each column indicate significant differences (*p* < 0.05) between treatments

The antiviral activity of AITC was assessed against HuNoV GI using ISC-RT-qPCR (Fig. 3). AITC at a concentration of 0.5%, incubated ON at 37 °C, decreased the binding ability of HuNoV to PGM by 43.7% compared to the control. This reduction was significantly higher than that observed with heat-treated HuNoV, which showed a 32.3% decrease of in PGM binding.

### Effectiveness of AITC as Natural Sanitizer on Vegetable Surfaces

To evaluate the potential use of AITC as a natural sanitizer, the surfaces of lettuce and spinach were treated with different concentrations (0.1 or 0.5%) of AITC for either 15 or 30 min. Tables 1 and 2 show the reductions of MNV and HAV titers on the inoculated vegetables. No significant differences were observed (*p* > 0.5) among the samples. The highest reductions were observed on lettuce surfaces (Table 1) inoculated with HAV, where, after 30 min of treatment at RT, AITC at concentrations of 0.1 and 0.5% reduced HAV titers by 0.71 and 0.81 log, respectively.

**Table 1:** Reduction of murine norovirus (MNV) and hepatitis A virus (HAV) titers (log TCID_50_/mL) on lettuce surfaces after 15 or 30 min AITC treatments at different concentrations at RT

Treatment time (min)	AITC (%)	MNV		HAV	
			Reduction		Reduction
15	0	6.03 ± 0.07a		5.99 ± 0.15a	
	0.1	5.87 ± 0.14a	0.17	6.03 ± 0.29a	− 0.04
	0.5	5.74 ± 0.19a	0.29	6.12 ± 0.19a	− 0.12
30	0	6.95 ± 0.45a		6.16 ± 0.14a	
	0.1	6.87 ± 0.58a	0.08	6.07 ± 0.12a	0.08
	0.5	6.62 ± 0.56a	0.33	5.95 ± 0.13a	0.21

Each treatment was done in triplicate

Different letters denote significant differences between treatments (*p* < 0.05)

**Table 2:** Reduction of murine norovirus (MNV) and hepatitis A virus (HAV) titers (log TCID_50_/mL) on spinach surfaces after 15 or 30 min AITC treatments at different concentrations at RT

Treatment time (min)	AITC (%)	MNV		HAV	
			Reduction		Reduction
15	0	3.82 ± 0.35a		5.16 ± 0.14a	
	0.1	3.82 ± 0.18a	0.00	4.74 ± 0.62a	0.42
	0.5	3.57 ± 0.18a	0.25	4.57 ± 0.22a	0.58
30	0	4.57 ± 0.00a		5.32 ± 0.00a	
	0.1	3.82 ± 0.71a	0.75	4.62 ± 0.51a	0.71
	0.5	3.76 ± 0.62a	0.81	4.54 ± 0.37a	0.81

Each treatment was done in triplicate

Different letters denote significant differences between treatments (*p* < 0.05)

## Discussion

The increasing frequency of foodborne virus outbreaks has heightened interest in alternative strategies for enhancing food safety and addressing health issues. Numerous natural compounds have been studied for their antibacterial properties, leading to investigations into their antiviral potential. Falcó et al. (2024) reviewed examples of such compounds, including green tea extract (GTE), caffeic acid (CA), algal extracts, grape seed extract (GSE), lignin, and curcumin. Additionally, the waste biomass of *Posidonia oceanica* has been valorised to produce extracts through different methodologies, with their antiviral properties have been evaluated (Benito-González et al., 2019). Similarly, by-products from the persimmon fruit have been evaluated as antiviral compounds and applied in the development of edible coatings on blueberries reducing MNV and HAV infectivity by 4.28 and 2.38 log, respectively, when polyphenol-rich pectin persimmon extracts with abundant non-covalent interactions were incorporated into the coating (Méndez et al., 2021, 2023).

Essential oils (EOs) and their derivatives represent another group of natural compounds that have been studied for their antiviral properties. Historically, industry has used EOs not only as flavoring agents but also as natural antimicrobials to improve food safety (Burt, 2004; Falcó et al., 2019). Although there are relatively few studies focusing on the antiviral activity of EOs or their components against enteric viruses, the existing findings suggest that their application in the food industry could be advantageous (reviewed by Falcó et al., 2024). Among the key compounds evaluated for their antiviral effects are carvacrol, zataria, lemongrass, allspice, mint, oregano, thymol, orange, grapefruit, and clove (Battistini et al., 2019; Chouhan et al., 2017; Elizaquível et al., 2013; Gilling et al., 2014; Kim et al., 2017; Kovač et al., 2012; Oh et al., 2013; Sánchez et al., 2015; Zhang et al., 2012). Significant antiviral activity has been reported for thyme, clove or allspice EO against HuNoV surrogates, with reductions exceeding a 3 logs (Chouhan et al., 2017; Fabra et al., 2016; Sánchez et al., 2015). However, findings on the efficacy of EOs against HAV have been inconsistent. For instance, thymol was tested by Sánchez and Aznar, (2015) without success, while Battistini et al. (2019) demonstrated the efficacy of lemon, grapefruit, and rosemary cineole EOs against HAV, resulting in nearly a 3-log reduction in viral titers.

In the present study, the application of AITC in suspension was successful in inactivating MNV and HAV in a dose-dependent manner, with higher concentrations of AITC leading to a greater reduction in viral titers. Additionally, the antiviral activity of AITC was affected by both contact time and temperature. For instance, incubating MNV with AITC at a concentration of 0.1% ON at 10 and 25 °C showed limited effects on viral concentration; however, at 37 °C, a significant reduction of 3.75 log was observed (Fig. 1). This temperature-dependent phenomenon has been reported in numerous studies under similar conditions. Mendez et al. (2023) investigated the impact of polyphenol-functionalized pectin derived from persimmon waste on viral inactivation at 37 °C. They found that both MNV and HAV levels dropped below the detection limit. At 25 °C, the highest reduction observed was 3.46 log for MNV, and approximately 2 log for HAV. Similarly, Randazzo et al. (2017) observed this temperature-dependent effect on enteric viruses treated with GTE, noting a reduction of more than 3 logs when comparing treatments at 4 °C to 37 °C. In the case of AITC, its high volatility likely plays a role, as temperature is known to influence both stability and release. Volatile compounds become less stable at higher temperatures, leading to more rapid degradation (Liu & Yang, 2010).

When treated with AITC at a concentration of 0.5%, more than a 2.5 log reduction in viral titers was recorded at different temperatures. These findings are in line with the antiviral properties of thymol EO (Battistini et al., 2019), where feline calicivirus (FCV) titers were reduced by over 3.5 logs. In our assays, MNV and HAV showed decreases of 3.17 and 2.37 logs, respectively, under the same concentration, exposure time, and temperature conditions. Similarly, our results reported only minimal reductions of 0.42 for MNV and 0.29 log for HAV after 2 h treatment at concentrations of 0.1 and 0.5%, respectively. This low efficacy has previously been reported with other essential oils under similar conditions. For instance, Chung (2017) reported log reductions of 0.48 and 0.64 for FCV and MNV, respectively, when using artemisia princeps var. oriental essential oil at concentrations of 0.1 and 0.01% after 1 h of treatment at RT. As anticipated, AITC showed higher inactivation rates for both viruses at higher concentrations compared to lower concentrations. This trend has been demonstrated in other studies, not only involving EOs, but also with various natural compounds, such as GTE, GSE or cinnamaldehyde (Joshi et al., 2015; Sánchez & Aznar, 2015).

Although partial success has been achieved in using human intestinal organoid cultures (HIOs) to cultivate infectious HuNoV in experimentally or naturally contaminated food and water samples (Carmona-Vicente et al., 2024; Estes et al., 2019; Falcó et al., 2023), the maintenance and differentiation of organoids remain time-consuming and costly, making them impractical for routine use. This limitation has significantly restricted the available information on the inactivation of infectious HuNoV.

Besides using norovirus surrogates, like MNV, Tulane virus, and FCV, alternative methods to measure infectious HuNoV have been reported. In the present study, ISC-RT-qPCR was used to determine the antiviral activity of AITC against HuNoV. PGM, which contains histoblood group antigens (HBGAs), has been recognized as receptors or co-receptors for HuNoV (Chung, 2017). In the previous research, ISC-RT-qPCR based on PGM was effectively utilized to assess the inactivation of HuNoV GI treated with different methods, including heat, high-pressure processing, GTE, chlorine, and ethanol (Falcó et al., 2018; Randazzo et al., 2017; Su & D’Souza, 2011). Our data suggest that 0.5% AITC inhibits the binding of HuNoV to the HBGAs found in PGM by 43.7%. This effect is comparable to that observed with aged-GTE at 0.5 mg/mL, which resulted in a 65% reduction in binding (Falcó et al., 2018).

In the fresh-cut vegetable sector, leafy greens pose a significant risk of cross-contamination (Moussaoui, 2013). The use of chlorine, the most commonly employed disinfectant, has been banned or limited in some European countries due to the formation of harmful chemical by-products, such as trihalomethanes (Battistini et al., 2019). As a result, in response to the growing demand for alternative disinfection methods, we evaluated AITC as a disinfectant for both lettuce and spinach leaf surfaces.

In the disinfection sanitation trials, AITC treatments did not statistically reduce viral contamination (Tables 1 and 2). Although in vitro testing has shown AITC to be effective against both viruses, this antiviral activity was not replicated when used as a sanitizer. Other compounds, including GTE, GSE, and carvacrol, have also been investigated as natural sanitizers (Sánchez et al., 2015; Wang et al., 2014). In our work, reductions of 0.75 and 0.81 for MNV and 0.71 and 0.81 for HAV were reported at concentrations of 0.1% and 0.5%, respectively. These findings are consistent with those of Su and D'Souza (2013), which reported reductions below 0.8 log for MNV after treating of lettuce with 1 mg/mL GSE. However, treatment of lettuce with 1% carvacrol for 30 min decreased MNV titers by 1.8 log (Sánchez et al., 2015). Given that AITC is a highly volatile molecule (Dancho et al., 2012; Tian et al., 2005), its antiviral potential in an open environment may be restricted for use as a natural sanitizer. AITC may be more effective in packaged foods, such as ready-to-eat foods or in food packaging materials. For instance, Seo et al. (2012) reported on the development of an AITC vapor releasing sachet, which demonstrated efficacy as an antimicrobial packaging system for fresh spinach. More recently, a study by Sharif et al. (2021) explored the antiviral properties of AITC in blueberry coatings, emphasizing its enhanced efficacy, especially at 37 °C. The study also explored the potential of agar and alginate-based coatings, with or without the antiviral extract, to inactivate enteric viruses (Fabra et al., 2018). Interestingly, while antiviral activity was greater at 37 °C, this effect was not dose-dependent. This can be attributed to the tendency of AITC to volatilize easily.

## Conclusion

In summary, this study highlights the potential of AITC as an effective natural compound against HAV and HuNoV, evaluated not only with surrogates but also using ISC-RT-qPCR for HuNoV. AITC demonstrated a concentration-, temperature-, and exposure time-dependent antiviral activity, achieving significant reductions in viral titers at higher concentrations and elevated temperatures. Although the in vitro studies demonstrated substantial inactivation of both viruses, the application of AITC as a sanitizer for vegetables was limited, likely due to its volatility in open environments.

The findings support further investigations into the application of AITC as a natural antiviral agent, particularly in packaged food setting or controlled environments where its antiviral properties can be maximized. Future research should focus on optimizing AITC application methods and exploring its effectiveness in real-world scenarios, such as fresh produce packaging or natural coating solutions. This study contributes to the growing body of evidence that natural compounds can serve as viable alternatives to traditional sanitizers, enhancing food safety and potentially reducing the risk of foodborne viral infections.

## Data Availability

All data were obtained from publicly available information.
